# Supplementary material for: Modeling glioblastoma heterogeneity as a dynamic network of cell states
Source: Mol Syst Biol. 2021 Sep 16;17(9):e10105. doi: 10.15252/msb.202010105 (PMC8444284; doi:10.15252/msb.202010105)
Supplement: Supplementary file 6 — Source Data for Figure 5 [file MSB-17-e10105-s004.zip › Figure5A_sourcedata/GSEA_3071/nosigdb_stateIII.GseaPreranked.1622795155562/DOROSZKO_PERIVASCULAR_INVASION.html]

Details for gene set DOROSZKO\_PERIVASCULAR\_INVASION[GSEA]

|  || Dataset | state33071 |
| Phenotype | NoPhenotypeAvailable |
| Upregulated in class | na\_neg |
| GeneSet | DOROSZKO\_PERIVASCULAR\_INVASION |
| Enrichment Score (ES) | -0.6989563 |
| Normalized Enrichment Score (NES) | -3.0445015 |
| Nominal p-value | 0.0 |
| FDR q-value | 0.0 |
| FWER p-Value | 0.0 |
Table: GSEA Results Summary

  

Fig 1: Enrichment plot: DOROSZKO\_PERIVASCULAR\_INVASION      
 Profile of the Running ES Score & Positions of GeneSet Members on the Rank Ordered List

  

| PROBE | GENE SYMBOL | GENE\_TITLE | RANK IN GENE LIST | RANK METRIC SCORE | RUNNING ES | CORE ENRICHMENT || 1 | S100A10 |  |  | 102 | 0.409 | -0.0001 | No |
| 2 | S100A6 |  |  | 172 | 0.360 | 0.0051 | No |
| 3 | PPFIBP1 |  |  | 462 | -0.252 | -0.0528 | No |
| 4 | RHOB |  |  | 865 | -0.272 | -0.1384 | No |
| 5 | BCHE |  |  | 867 | -0.272 | -0.1213 | No |
| 6 | KLF4 |  |  | 973 | -0.278 | -0.1305 | No |
| 7 | ETV1 |  |  | 1495 | -0.306 | -0.2445 | No |
| 8 | DAG1 |  |  | 1598 | -0.312 | -0.2508 | No |
| 9 | PGM2L1 |  |  | 2260 | -0.354 | -0.3975 | No |
| 10 | PCSK1N |  |  | 3346 | -0.470 | -0.6454 | No |
| 11 | DKK1 |  |  | 3556 | -0.530 | -0.6652 | Yes |
| 12 | CKB |  |  | 3572 | -0.536 | -0.6349 | Yes |
| 13 | NES |  |  | 3596 | -0.545 | -0.6061 | Yes |
| 14 | MAP2 |  |  | 3640 | -0.572 | -0.5807 | Yes |
| 15 | GNG7 |  |  | 3722 | -0.633 | -0.5612 | Yes |
| 16 | LMO4 |  |  | 3734 | -0.650 | -0.5226 | Yes |
| 17 | VCAN |  |  | 3741 | -0.660 | -0.4821 | Yes |
| 18 | F3 |  |  | 3764 | -0.693 | -0.4436 | Yes |
| 19 | COL6A1 |  |  | 3863 | -0.872 | -0.4132 | Yes |
| 20 | GAS7 |  |  | 3871 | -0.933 | -0.3557 | Yes |
| 21 | TNC |  |  | 3874 | -0.951 | -0.2956 | Yes |
| 22 | PTN |  |  | 3884 | -1.022 | -0.2329 | Yes |
| 23 | IGFBP5 |  |  | 3895 | -1.082 | -0.1666 | Yes |
| 24 | PTPRZ1 |  |  | 3913 | -1.342 | -0.0855 | Yes |
| 25 | IGFBP2 |  |  | 3917 | -1.404 | 0.0031 | Yes |
Table: GSEA details [plain text format]

  

Fig 2: DOROSZKO\_PERIVASCULAR\_INVASION: Random ES distribution      
 Gene set null distribution of ES for **DOROSZKO\_PERIVASCULAR\_INVASION**
